# Metabolomics: moving towards personalized medicine

**DOI:** 10.1186/1824-7288-35-30

**Published:** 2009-10-23

**Authors:** Eugenio Baraldi, Silvia Carraro, Giuseppe Giordano, Fabiano Reniero, Giorgio Perilongo, Franco Zacchello

**Affiliations:** 1 Department of Pediatrics, University of Padova, Padua, Italy; 2 European Commission, Joint Research Centre, Institute for Health and Consumer Protection, Physical and Chemical Exposure Unit, Ispra (VA), Italy

## Abstract

In many fields of medicine there is a growing interest in characterizing diseases at molecular level with a view to developing an individually tailored therapeutic approach. Metabolomics is a novel area that promises to contribute significantly to the characterization of various disease phenotypes and to the identification of personal metabolic features that can predict response to therapies. Based on analytical platforms such as mass spectrometry or NMR-based spectroscopy, the metabolomic approach enables a comprehensive overview of the metabolites, leading to the characterization of the metabolic fingerprint of a given sample. These metabolic fingerprints can then be used to distinguish between different disease phenotypes and to predict a drug's effectiveness and/or toxicity.

Several studies published in the last few years applied the metabolomic approach in the field of pediatric medicine. Being a highly informative technique that can be used on samples collected non-invasively (e.g. urine or exhaled breath condensate), metabolomics has appeal for the study of pediatric diseases. Here we present and discuss the pediatric clinical studies that have taken the metabolomic approach.

## Introduction

Many multifactorial diseases may have a homogeneous clinical presentation but still be heterogeneous from a molecular standpoint and the concept that every patient's disease is somehow unique is gaining ground, in which case a customized, individually-targeted therapy would be desirable [1]. For the development of such a personalized therapeutic approach to be feasible, diseases need to be defined and characterized at molecular level [1]. Recent advances in high-throughput technologies (e.g. mass spectrometry and NMR-based spectroscopy) have enabled the identification of biomarker profiles that characterize disease sub-phenotypes, providing the basis for the development of new, targeted drugs, potentially enabling us to provide "the right therapy for the right patient" [1]. It is on such technologies that the so-called "-omic" sciences rely, e.g. genomics, transcriptomics, proteomics, metabolomics, where these "-omic" terms have been formulated to define approaches capable of identifying groups of biomarkers characteristic of a particular disease within the set of genes, mRNAs, proteins and metabolites of a given organism [2,3].

Metabolomics is defined as the analysis and interpretation of the global metabolic data expressing the multiparametric metabolic response of living systems to genetic modification, pathophysiological stimuli and environmental influences [4,5]. Metabolomics takes a non-selective approach and, by means of a comprehensive overview of the metabolites, enables the "metabolic fingerprint" of a sample to be obtained [6]. Metabolomics is the latest of the -omic sciences and it is considered the one that comes closest to expressing phenotype, giving us the chance to look at genotype-phenotype, as well as genotype-envirotype relationships. In fact, although the metabolic profile can be seen as the ultimate expression of the information contained in the genetic code, it is also influenced by several factors unrelated to the genome, such as interactions with commensal microorganisms, nutritional factors, environmental agents, and exposure to drugs or toxic substances [3,6].

From a clinical standpoint, the metabolomic analysis has two major potential applications. The first concerns the early diagnosis and characterization of disease phenotypes. Metabolomic analysis can detect a pattern of metabolites that discriminate between groups of subjects, enabling the metabolic characterization of a disease, or of a disease phenotype. This is an exploratory process, since unexpected or even unknown metabolites may turn out to be important in this discrimination, paving the way to the formulation of new pathophysiological hypotheses [2,4].

The second potential clinical application concerns the identification of individual metabolomic characteristics able to predict drug effectiveness and/or toxicity - an approach called pharmacometabolomics, which appears to be a promising branch of metabolomics for screening human populations, implying the concrete possibility of a genuinely customized approach to treatment [1]. The concept of pharmacometabolomics has been first introduced by a seminal study conducted on mice demonstrating that the hepatotoxic effects of the analgesic paracetamol can be predicted on the basis of the pre-treatment metabolomic urinary profiles [7].

## Methods used in metabolomic analysis

The methods used in metabolomic analysis are generally based on mass spectrometry or NMR-based spectroscopy, since these techniques can handle complex biological samples with a high sensitivity, selectivity and throughput [8]. Mass spectrometry, usually combined with chromatographic separation methods, enables the molecules in a sample to be separated on the basis of their mass-to-charge ratio and their representation in a spectrum [9,10].

^1^H-NMR spectroscopy enables the detection of almost all proton-containing metabolites in a sample, different molecules producing different signals in the NMR spectrum [11]. Some of the advantages of this technique are that it is non-selective and fast, and usually demands no sample preparation [11]. It is also non-destructive and can be applied to the analysis of tissue samples that will subsequently remain available for further diagnostic analyses [11].

A recent development is represented by the combination of NMR and MS data, which may improve the identification of unknown metabolites [12].

Both NMR and MS are powerful spectroscopic methods for generating multivariate datasets: NMR and MS spectra are highly complex and the biological information they contain can only be extracted by applying bioinformatic tools, such as pattern recognition methods. These are computer-based procedures that can be classified as unsupervised or supervised [13]. The unsupervised methods reduce the complexity of the data contained in the spectra and represent them by means of plots that the human eye can interpret. This approach helps to identify any intrinsic sample clustering, to see whether different groups of individuals (e.g. healthy vs ill) can be discriminated by the characteristics of their spectra [13]. The supervised methods use a training set of samples (of known classification) to create a mathematical model that is then used to test an independent dataset: unlike the unsupervised methods, they enable us to predict which group a new sample belongs to on the strength of the characteristics of its spectra [13].

Once a metabolic pattern typical of a given condition has been characterized, the analysis may go on to identify single biomarkers relevant to sample clustering. Fundamental support for molecular identification comes from various on-line databases, the most comprehensive of which is the Human Metabolomic Database [14].

## Pediatric clinical studies

Metabolomic analysis can be applied to the study of biological fluids collected in non-invasive or minimally-invasive ways (e.g. urine, exhaled breath condensate, blood). Being highly informative and suitable for use on non-invasively collected samples too, the metabolomic approach seems particularly promising in the field of pediatric medicine [15].

A number of recently-published studies have applied the metabolomic approach to the pediatric population. Some of these studies evaluated how physiological variables, such as age or diet, can affect children's metabolomic profiles.

Using an NMR-based metabolomic analysis, Gu et al demonstrated the effect of age on the urinary metabolite profile in pre-adolescent children [16]. Likewise, a recent Italian study evaluated urine samples in genetically homogeneous healthy populations, demonstrating that the excretion of most amino acids is age-dependent [17]. Understanding the age-related characteristics of the metabolomic profile can facilitate the interpretation of its pathological modifications [16].

To study the effects of diet, Bertram et al [18] compared the metabolomic urinary profiles of two groups of children on a diet rich in milk versus meat proteins, demonstrating that the samples could be successfully discriminated according to the children's diet, with a significantly greater urinary excretion of creatine in the children on a diet rich in meat.

Other pediatric studies have focused on the metabolomic profiles of different biofluids in the presence of pathological processes.

In one publication, metabolomics was used to study disorders due to inborn errors of metabolism [19]. Mass spectrometry is commonly used in the diagnosis of inborn errors of metabolism to seek specific metabolites (targeted analysis), but this study was the first to use untargeted metabolomic analyses on plasma samples. Investigating disorders of proprionate metabolism (methylmalonic acidemia [MMA] and propionic acidemia [PA]), the authors found that the most important metabolite for discriminating between the healthy and the ill was propionyl carnitine, which is indeed the target compound for screening newborn for MMA and PA by tandem-MS. This result validates the role of untargeted metabolomic analysis in identifying biomarkers of disease. In addition, untargeted metabolomic analysis showed that many other compounds are important in differentiating both between healthy and ill, and between cases of MMA and PA [19].

A Japanese group described a characteristic metabolomic profile in the cerebrospinal fluid of children with influenza-associated encephalopathy, suggesting that it might be possible to identify specific biomarkers useful for the early diagnosis of this disease [20].

In a study by our group [21], metabolomics was applied to analyzing exhaled breath condensate (breathomics): this biofluid is collected non-invasively by cooling the exhaled air and its composition is believed to reflect that of the airway lining fluid [21]. NMR-based metabolomic analyses of exhaled breath condensate could clearly discriminate between asthmatic and healthy children, with 95% success rate in their classification. Many authors believe that asthma should no longer be considered a single disease and that efforts should be made to identify the different biochemical and inflammatory profiles behind asthma symptoms in order to treat them with specifically-targeted therapies [22]. The metabolomic approach may have a role in characterizing these asthma sub-phenotypes.

Still in the field of pediatric pulmonology, it has recently been demonstrated that metabolomic analysis of bronchoalveolar fluid can distinguish between different degrees of inflammation in children with cystic fibrosis, with the promise of identifying new biomarkers of inflammation [23].

Metabolomic analysis has also proved useful in identifying early urinary biomarkers of acute kidney injury after cardiopulmonary bypass surgery [24]: using ultra-performance liquid chromatography (UPLC)/MS based metabolomic analyses on urine samples collected 4 and again 12 hours after surgery, the authors were able to identify the children that would develop acute kidney injury over the next 3 days. From a clinical standpoint, finding early metabolic biomarkers may improve our understanding of the pathophysiological mechanisms involved in acute kidney injury and enable a timely diagnosis of this disease after pediatric cardiac surgery.

Finally, an interesting longitudinal study applying the metabolomic analysis to serum samples recently demonstrated that a metabolic dysregulation precedes the onset of the autoimmunity associated with type-1 diabetes and the following progression to clinical diabetes [25]. This study has potential therapeutic implications too: based on their findings, the authors suggest that an immunomodulatory therapy might be more useful than immunosuppression in the prevention of the disease [25].

## Conclusion

Metabolomics is a novel approach that promises to enable the detection of states of disease, to stratify patients based on biochemical profiles and to monitor disease progression. Metabolomic analysis may also be able to orient the choice of therapy, identify responders and predict toxicity (pharmacometabolomics), paving the way to a customized therapy.

There are many potential applications for metabolomics in pediatric medicine, inasmuch as it is a highly informative technique that can also be used on non-invasively collected samples.
